# Surgical challenge: endoscopic repair of cerebrospinal fluid leak

**DOI:** 10.1186/1756-0500-5-459

**Published:** 2012-08-27

**Authors:** Carlos Martín-Martín, Gabriel Martínez-Capoccioni, Ramón Serramito-García, Federico Espinosa-Restrepo

**Affiliations:** 1 Servizo Galego de Saúde. Service of ENT–Head and Neck Surgery, University Hospital Complex of Santiago de Compostela (CHUS), Santiago de Compostela, Spain; 2 Servizo Galego de Saúde. Service of ENT–Head and Neck Surgery, Hospital da Barbabanza, La Coruña, Spain; 3 Servizo Galego de Saúde. Service of Neurosurgery, University Hospital Complex of Santiago de Compostela (CHUS), Santiago de Compostela, Spain

**Keywords:** Cerebrospinal fluid leak, Endoscopic sinus surgery, Anterior skull base, Bone defect, Bacterial meningitis

## Abstract

**Background:**

Cerebrospinal fluid leaks (CSF) result from an abnormal communication between the subarachnoid space and the extracranial space. Approximately 90% of CSF leak at the anterior skull base manifests as rhinorrhea and can become life-threatening condition. Endoscopic sinus surgery (ESS) has become a common otolaryngologist procedure. The aim of this article is to consider our experience and to evaluate the outcomes in patients who underwent a purely endoscopic repair of CSF leaks of the anterior skull base.

**Findings:**

Retrospective chart review was performed of all patients surgically treated for CSF leaks presenting to the Section of Nasal and Sinus Disorders at the Service of ENT–Head and Neck Surgery, University Hospital Complex of Santiago de Compostela (CHUS), between 2004 and 2010. A total of 30 patients who underwent repair CSF leak by ESS. The success rate was 93.4% at the first attempt; only two patients (6.6%) required a second surgical procedure, and none of it was necessary to use a craniotomy for closure. Follow-up periods ranged from 4 months to 6 years.

**Conclusion:**

Identifying the size, site, and etiology of the CSF leak remains the most important factor in the surgical success. It is generally accepted that the ESS have made procedures minimally invasive, and CSF leak is now one of its well-established indications with low morbidity and high success rate, with one restriction for fistulas of the posterior wall of the frontal sinus should be repaired in conjunction with open techniques.

## Findings

### Introduction

Cerebrospinal fluid leaks (CSF) result from an abnormal communication between the subarachnoid space and the extracranial space**.** CSF leaks of the anterior skull base present one of the more difficult challenges in Endonasal edoscopic surgery (EES), involving an area that is anatomically complicated and technically demanding to access. The challenge is to recreate the barrier between the cranial vault and the nasal cavity to prevent and eliminate cerebrospinal fluid (CSF) leaks and protect the brain from exposure to infectious sources. Approximately 90% of CSF leaks at the anterior skull base manifests as rhinorrhea and can become life-threatening condition [1,2]. CSF leaks bears the risk of meningeal or intracranial infection and complication [3]. And any persistent CSF leak should be repaired [1]. Evaluation of the endoscopic repair of sinonasal CSF leaks has shown high success rates of 90% for first attempts at repair and up to 97% following a second endoscopic repair [1-3].

ESS has become a common otolaryngologist procedure. EES is a minimally invasive surgical technique, which provided a direct short-cut access to anterior and middle skull base without traversing any mayor neurovascular structures, which until recently accounted for a significant morbidity and a high-risk surgery [4] and CSF fistula is now one of its well-established indications.

CSF leaks can have many etiologies including spontaneous, tumor-related, traumatic (accidental or iatrogenic), or congenital leaks. Etiology affects the risk of recurrence and thus the method of repair by having an impact on the defect size, location, degree of dural involvement, the likelihood of elevated intracranial pressure (ICP), and the possibility of meningoencephalocele protrusion [5-7].

The aim of this article is to consider our experience and to evaluate the outcomes in patients who underwent a purely endoscopic repair of CSF leaks of the anterior skull base.

## Methods

Retrospective chart review was performed of all patients surgically treated for CSF leaks of the anterior skull base presenting to the Section of Nasal and Sinus Disorders at the Service of ENT-Head and Neck Surgery, University Hospital Complex of Santiago de Compostela (CHUS), between 2004 and 2010. Data were collected according to the patient’s characteristics, CSF fistula, surgical techniques, materials used to repair the defect, and complications. We will consider the 30 patients who underwent repair CSF leaks by ESS. 16 (53,3%) men and 14 (46,6%) women (aged between 29 and 60 years, median age 41.2 years). The study was approved by the University Hospital Complex of Santiago de Compostela (CHUS) medical ethics board.

### Preoperative evaluation

Preoperatively patients underwent various evaluations to confirm CSF leak and location, including thorough history and physical with nasal endoscopy and testing including magnetic resonance imaging (MRI), computed tomography (CT), β-trace testing and β-2 transferrin testing for confirmation of CSF rhinorrhea (a protein found almost exclusive in CSF, perilymph in the cochlea and the aqueous and vitreous humor of the eye, so it has high sensitivity and specificity, and have high false-positive rate in cases of cirrhosis or hereditary protein anomalies). In cases where the leak had stopped we relied on high resolution CT scan and CT cisternography to determine the leak and location. In three patients with ascending bacterial meningitis the high resolution CT and endoscopic examination showed the location of the bone defect.

### Surgical technique

The precise location of site and size of fistula is the keystone for successful endoscopic closure and closure of the leak was performed by EES. The closure techniques depend on the size and location of cranial defect, the three forms of grafting are the underlay, overlay and combined. “Underlay” or “inlay” technique the intact dura is separated from the edge of the skull base defect to expose an adequate buttress for the stabilization of the graft. The free graft, or flap, should be designed in such a way that it can be pushed a few millimeters between the bone and the dura on all sides of the defect.

Bone or cartilage underlay grafts are advocated for large, bony defects associated with herniating brain or meninges. “Overlay” or “onlay” technique (the graft is placed generally over the dural lesion and over the exposed bony margins, which have been denuded of mucosa.), is a technique for small defects. The third approach is to place two separate grafts, one as an overlay and the other as an underlay.

The size of the defect also has an impact on surgical planning for the type of grafting required, as smaller defects are more conducive to pliable overlay grafts, and larger (>3 mm) sites can accommodate an underlay graft or multilayer closure with both underlay and overlay grafts. Other options for situations requiring a stronger reconstruction (very large defects or elevated ICP that could dislodge a soft graft) include bony underlay grafts and soft overlay grafts with bony countersinking techniques [8-10].

## Results

**Figure 1:**
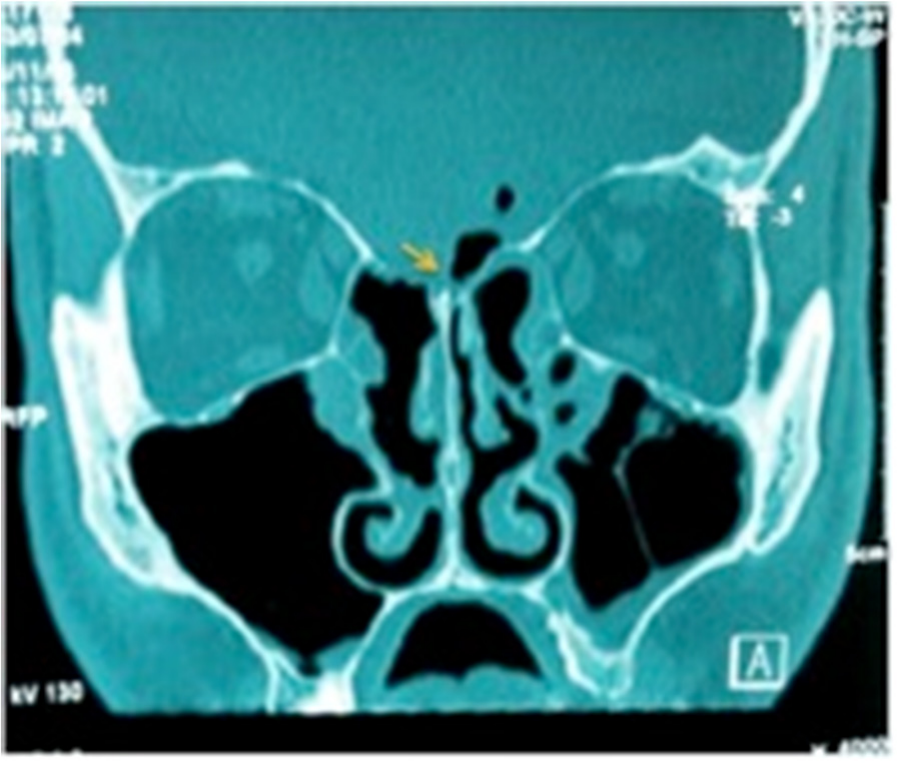
**Postoperative pneumocephalus following FESS.** Coronal CT scan demonstrates a minimal amount of pneumocephalus (Yellow arrow) and defect in the left ethmoid roof adjacent to the middle turbinate insertion following FESS.

**Figure 2:**
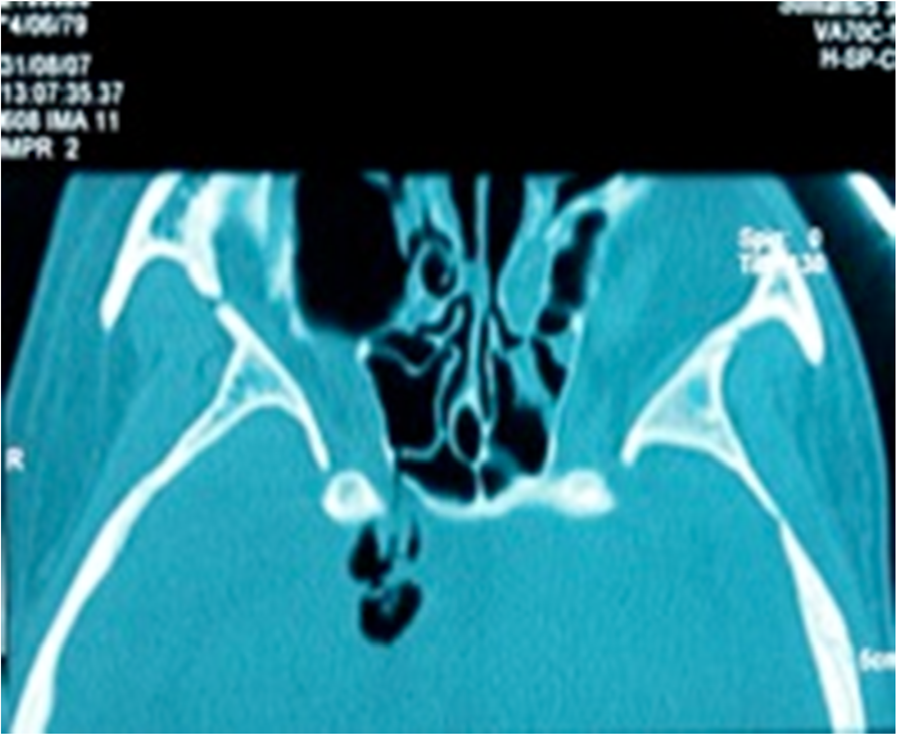
**Postoperative pneumocephalus following septum and turbinate surgery.** Axial CT scan demonstrates a minimal amount of pneumocephalus (Yellow arrow) and defect in the right sphenoidal sinus.

From the etiological point of view, dural defects were classified into three groups:

Traumatic (20, 66.6%): 12 (40%) were iatrogenic CSF fistula, these patients had acquired leaks following traditional sinonasal surgical approaches or functional endoscopic sinus surgery (FESS) and 8 (26,6%) patients were caused by closed head injury. The iatrogenic (40%): 9 (75%) patients by FEES (Figure 1), 2 (16,6%) patients for removal a meningioma and 1 (8,3%) patient after septum and turbinate surgery (Figure 2). Of these, the CSF leaks were caused by the use of microdebrider blade in 3 (25%) patients, being identified and closed immediately in the same surgical procedure. In 6 (50%) patients the CSF leak was diagnosed after surgery, because they developed serious complications, they suffered bacterial meningitis and two developed a pneumocephalus (Figures 1 and 2). The closure of the CSF leak was made after resolving infectious pathology.

**Figure 3:**
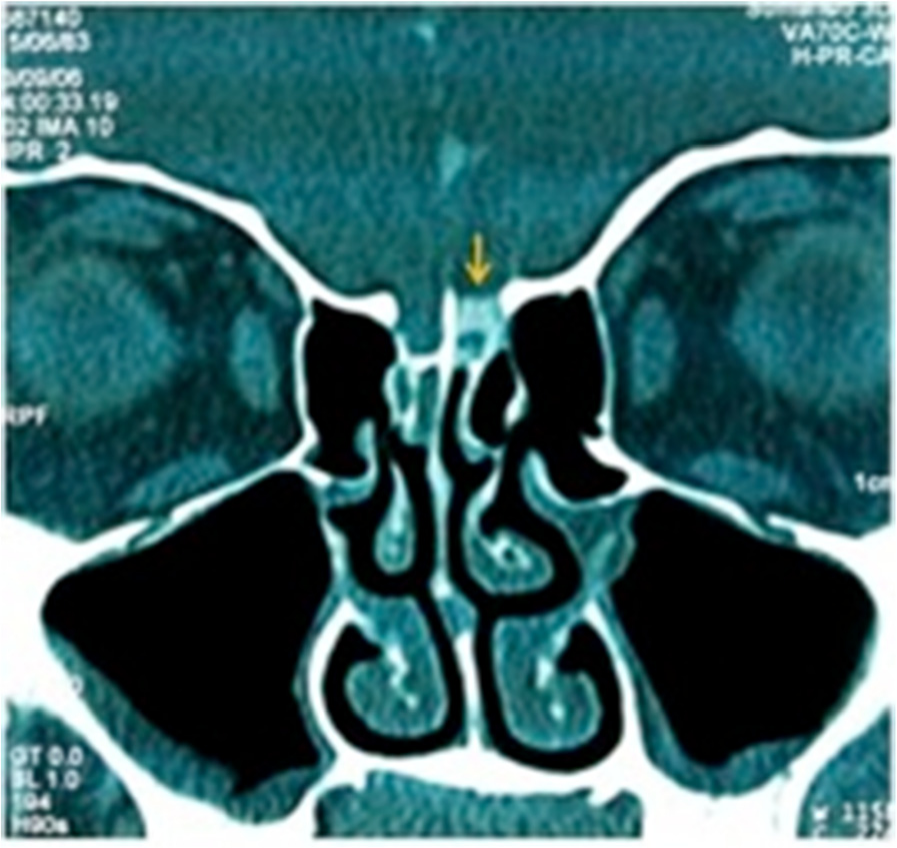
Coronal CT in a patient with a spontaneous CSF leak in etmoid roof.

Nontraumatic (8, 26.6%): this group included adults without any previous history of trauma or surgery (Figure 3).

Tumor invading the skull base (2, 6,6%) patients: a sinus tumor invading the skull base and debuted with a CSF leaks.

The most frequent site of the anterior skull base defect was the cribriform plate (23,4%), followed by the ethmoid roof (76,6%).

The closure technique used depended on the size and location of the fistula. We use the used the underlay technique in 20 (66,6%) patients. The onlay technique was used in 10 (33,4%) patients. The most frequently used graft was mucoperichondrium combined with cartilage harvested from the nasal septum harvested either alone (18, 60%), or mucoperichondrium either alone (12,40%).

The success rate was 93.4% at the first attempt; only two patients (6.6%) required a second surgical procedure, and none of it was necessary to use a craniotomy for closure. Follow-up periods ranged from 4 months to 6 years.

Confirmation of CSF at the nasal secretion was demonstrated by quantification of β2-transferrin testing in 24 (80%) patients and β-trace testing in 8 (26,6%). The radiological diagnosis by CT cisternography confirmed the CSF leaks and location the defect in 18/30 (60%) patients. In 6 (20%) patients was not necessary because the fistula was identified and closed intraoperatively and the remaining 6 patients with bacterial meningitis, the high resolution CT and endoscopic examination showed the location of the bone defect.

The leak size was determined by high resolution CT and measurement the defect with curettes in the surgical field, in 10 (33,4%) patients was less than 3 mm, and 20 (66,6%) patients greater than 3 mm.

In all cases we use fibrin glue to improve adherence of the graft, and the graft is supported in place with layers of Surgicel®, to separate the graft from the packing material, to prevent avulsion of the graft or flap during its removal. One or two pieces of Merocel packs (XOMED, Jacksonville, Fla) into a sterile glove finger were placed in the nasal cavity at the end of the procedure. Packs were removed from the common nasal meatus on the third day. We use ceftriaxone as antibiotic coverage during the hospitalization period.

The post-operative phase, conservative measures such as bed rest, avoidance of straining and Valsalva maneuvers, especially vomiting, blowing the nose or avoiding uneasy defecation, can be targeted as ways of avoiding rapid changes in intracranial pressure. The slow resumption of normal activities is mandatory. A post-operative follow-up visit is arranged at 1–2 weeks, with conservative management of crusting. Debridement is undertaken only at 3–4 weeks postoperatively to minimize the possibility of dislodging the graft. Regular follow-up continues weekly until the repair leak site is completely mucosalised and ventilation of paranasal sinuses is ensured.

In 2 (6,6%) patients (the first two that were operated in our department) underwent lumbar puncture before surgery, which was intended to ensure that non-surgical closure of a seal failed. And in other 2 (6,6%) patients, lumbar puncture took place at the end of surgery by a large surgical defect. In others patients, the lumbar puncture was avoided when considering major postoperative morbidity associated and the conservative measures.

## Discussion

In recent years we have seen the establishment of the ESS as a technique of choice for closure of CSF leak, seen as a less invasive technique with less morbidity and mortality, excellent view of the surgical field, and a higher success rate (about 95%), replacing the usual techniques, such as transcranial and extracranial interventions that had a success rate of 70% with significant morbidity (anosmia was permanent sequel) [9]. It is a common surgical approach in the surgeon’s nose that does not require specific instruments for intervention. However, it is necessary to get a good workout in endonasal endoscopic techniques in order to obtain good surgical results and and avoid complications. And one way to achieve these objectives is the organization of neurosurgeons and otolaryngologists teams to share experiences and knowledge to the management of skull base pathology [10].

The presence of CSF rhinorrhea entails a significant risk to the patient’s life [3]. The clinical confirmation should be performed by nasal inspection and determination of CSF markers such as β2-transferrin, which has high specificity and sensitivity [11], or β-trace protein, economical and highly specific with high sensitivity. A CSF fistula entails a risk of bacterial meningitis in the long term, approximately 40% [12], situation may worsen when the healing of the repair is insufficient, when you leave a lumbar drain, or the administration of prophylactic antibiotics that increase the bacterial resistance and promote infection [3].

The pneumocephalus is other major complication in the anterior skull base defects, and are due to direct contact between nasal cavity and the intracranial cavity. The rapid onset of headache and neurological signs, changes in mental status to coma should alert. The two patients who had a severe pneumocephalus, CT confirmed the presence of intracranial air and the fistulous tract through the ethmoid roof into the nasal cavity. It is caused by either a ball-valve mechanism that allows air to enter but not to exit, or by CSF leakage, which creates a negative pressure with subsequent air entry [13]. The closures of both fistulas were performed with an underlay technique, placing a mucoperichomdrium combined with cartilage graft, and seal with fibrin glue without lumbar puncture. The follow-up CT certified the progressive resolution of pneumocephalus confirmed the closure of CSF leaks.

The identification of the site is necessary for successful surgical repair. CT, with and without contrast, and nasal endoscopic exploration are the most common form of locating the fistula, and when not displayed properly, CT cisternography is a helpful test [14].

In the surgical iatrogenic CSF fistula, need to clean and control the nasal bleeding that occurs in the surgical field to locate and properly close the fistula. It should be noted the risk that exists with the use of microdebrider blade in FESS, due to the fast and aggressive cut that exposes the skull base to iatrogenic injury, in this area is common to use less aggressive material but we must remember that the skull base lesions can occur with any instrument or technique [1]. We noted in one patient a large iatrogenic injury at the ethmoid region after endoscopic nasal surgery as a result of using a microdebrider blade, which was repaired in a second attempt. In general, iatrogenic CSF fistula after FESS are small, and as previously mentioned, the size of the defect is the factor determinant, for the need the additional layers and supporting structures.

The observation in one of our patients with spontaneous CSF fistula the presence of idiopathic intracranial hypertension, has led us to consider the need for a previous study as the search for an empty sella syndrome [15]. The presence of obesity by body mass index [10,16], or observation intracranial hypertension by ophthalmologic study.

There have been many materials used for sealing of the fistula, and we have resorted to many. The mucoperichondrium, and cartilage in our patients were able to seal the fistula, and we agree with Hegazy [6], which reports that the material used in the closure of the fistula is not important in the success of the intervention, even in large defects of choosing a suitable material is important [17]. Most authors recommend obtaining grafts of the nasal passages that can be easily obtained from the turbinates, nasal septum or nose floor, but if you cannot get, the temporalis fascia is still the most appropriate place. Either way, each fistula should be treated in a unique way [18], and the surgeon must know the different options to solve the problem.

The controversy lies in the technique of graft placement. Onlay and underlay techniques are used depending on the size of the fistula, and both have similar results when used properly [19]. Determine the size of the fistula is important. For this we use curettes of different sizes than we use in pituitary surgery trying, to cut the graft to form double defect. If necessary, use a layer of septal cartilage to provide better management when inserted into the defect. We prefer to perform the underlay technique, as a safe technique, because the base of the skull is that supports the graft in place. In addition, to prevent a brain herniation, place a piece of cartilage that gives it strength to clogging with perichondrium. In small leak where it is technically impossible “underlay” technique, we perform the onlay technique. It is widely accepted that large defects are preferably treated by underlay technique, and we recommend them to be placed into the fistula by a piece of cartilage, which can be obtained from the nasal septum. This gives strength to the graft of perichondrium and avoids brain herniation. It is important to promote osteogenesis curettage is performed with a curettes of bone defect edges that we will close. Onlay techniques is reserved for small defects, or when the underlay technique is not possible.

We do not use intrathecal fluorescein for serious complications that can arise. We believe that lumbar drainage may increase morbidity and hospital stay. To observe the leak during surgery, we encourage increased intracranial pressure by increasing the pressure at the abdomen that allows us to locate the CSF leak as a stream of clear liquid and transparent.

Like others authors [17,20], we reserve lumbar drainage for patients with elevated intracranial pressure and the conservative measures, such as bed rest, elevation of the head, avoidance of straining activities [10], is sufficient to ensure the sealing of the leak. An area of controversy regarding management involves the use of CSF diversion techniques such as lumbar drainage. Some authors hypothesize that regardless of the reconstruction technique, patients with increased CSF pressure are at increased risk of persistent or recurrent CSF leak at the reconstruction site or elsewhere along the skull base. While some groups do not favor the use of perioperative lumbar drainage because closure rates may not improve and fear of eliciting pneumocephalus [21-23] others use lumbar drainage to measure intracranial pressure to select patients for permanent CSF diversion [5].

The use of antibiotics in skull base surgery is controversial, however the penicillin and macrolides are used in the postoperative phase of endoscopic sinus surgery, and although the risk of meningitis must be counterbalanced with the risk of resistance to antibiotics, we recommend an antibiotic coverage in cases of iatrogenic fistulas. Like other authors we recommend the use of ceftriaxone [15]. Hospitalization should be extended only the time that patient is a monitored and intravenous antibiotic, although some authors recommend the patient was discharged one day after the intervention [19].

## Conclusion

The control of CSF leakage has been significantly improved through the development of the EES. The excellent exposure of the nasal cavity roof by endoscope offers the opportunity to identify the area of the fistula and allowing an adequate treatment plan. It is generally accepted that the ESS have made procedures minimally invasive, and (CSF) fistula is now one of its well-established indications with low morbidity and high success rate, with one restriction for fistulas of the posterior wall of the frontal sinus should be repaired in conjunction with open techniques.

Identifying the size, site, and etiology of the CSF fistula remains the most important factor in the surgical success. The risk of bacterial meningitis, with a significant mortality rate, it’s high enough to consider surgical closure of the fistula. Provides excellent results, and allows us to address a problem that until recently was a serious medical conflict.

## Competing interests

The authors declare that they have no competing interests.

## Authors’ contributions

CM-M, GM-C, RS-G and FE-R designed the study. CM-M coordinated sample and medical record data collection. All authors contributed to writing and reviewing the final manuscript. All authors read and approved the final manuscript.
